# Crowdsourced audit of Twitter’s recommender systems

**DOI:** 10.1038/s41598-023-43980-4

**Published:** 2023-10-05

**Authors:** Paul Bouchaud, David Chavalarias, Maziyar Panahi

**Affiliations:** 1 CNRS, Complex Systems Institute of Paris Île-de-France (ISC-PIF), 75013 Paris, France; 2 EHESS, Center for Social Analysis and Mathematics (CAMS), 75006 Paris, France

**Keywords:** Computer science, Computational science, Information technology

## Abstract

This research conducts an audit of Twitter’s recommender system, aiming to examine the disparities between users’ curated timelines and their subscription choices. Through the combined use of a browser extension and data collection via the Twitter API, our investigation reveals a high amplification of friends from the same community, a preference for amplifying emotionally charged and toxic *tweets* and an uneven algorithmic amplification across friends’ political leaning. This audit emphasizes the importance of transparency, and increased awareness regarding the impact of algorithmic curation.

## Introduction

Social media platforms like Twitter have become integral to modern communication, facilitating the exchange of information and ideas on a global scale. Within these platforms, the central gateway for users navigating the vast amount of user-generated content is the *Newsfeed*, which is thoughtfully curated to maximize user engagement. These systems, which filter content from one’s social environment, function as attention-allocators^1^, wielding substantial influence over the content encountered by users and thereby present a potential systemic risks to society. A notable study by Huszár et al^2^ demonstrated how Twitter’s recommender system amplified the reach of political *tweets* depending on their ideological alignment. To demonstrate this, they leveraged proprietary data on Twitter users and conducted a years-long experiment, which included a controlled group of nearly two million users not exposed to Twitter’s recommender system. For lack of direct access to data, independent audits of social media recommender systems has been addressed mostly through so-called “sock-puppet audit”, creating artificial users and scrapping the platform content. While providing interesting insights into the algorithmic curation, such audits are limited by the number of fake accounts that researchers can create —^3,4^ used 8 accounts in their demonstration— and their ability to realistically mimic human digital behavior —usually ad hoc heuristics. Enlisting volunteers to provide their data —made more easily accessible to them in recent years thanks to legislative progress such as with the GDPR— seems to gradually become a promising avenue in digital services independent external audits^5–7^. Yet, the relative lack of control is a common drawback of a purely crowdsourced audits.

To overcome these challenges, we adopted a dual approach involving the use of a desktop browser extension to capture Twitter *feed* content from volunteers and an extensive data collection through the Twitter API. This methodology allowed us to compile a set of messages authored by participants’ friends; pool of messages that Twitter’s recommender system curated to compose participants’ timelines. Our goal is to compare the information landscape portrayed by the recommender system and user’s subscription choices, without claiming to comprehensively investigate the intricate socio-technical aspects or unravel feedback loops^8^. In particular, our findings reveal that Twitter’s recommender system: (i) significantly amplifies content authored by small or quiet accounts; (ii) unevenly amplifies *tweets* from users’ friends based on their political leaning, portraying a political landscape distinct from users’ subscription choices; (iii) greatly elevates the visibility of friends belonging to the same community as the user; and (iv) amplifies toxic and emotionally charged *tweets* while reducing the visibility of neutral ones.

## Methods

### Context

Our analysis was conducted prior to the release of the overall architecture and partial source codes of Twitter’s recommender systems on March 31, 2023, a preliminary pre-print can be found at hal-04036232v2. The current analysis has been re-performed over a more recent timeframe, specifically 07/03/23-06/04/23 for account-level features and 14/01/23-07/02/23 for tweet-level features, as explained below. Among the numerous hard-coded heuristics and general insights, Twitter engineers have specifically highlighted the significance of community detection in the recommendation process^9^. In light of this, we have conducted an additional analysis to examine whether *tweets* from friends belonging to the same community as the participant are amplified compared to those from different communities.

### Data collection

We developed a browser-extension called “Horus”, compatible with Chrome and Firefox, that allows us to capture various data, including Twitter feeds, displayed on participants’ desktop screens. The participants were self-selected, they chose to take part in the study after becoming aware of it through newspaper articles and radio broadcasts in the fall of 2022. To expand our reach and attract a wider range of individuals, we also employed online advertising on Twitter, presenting the initiative and encouraging individuals to participate. In addition to contributing to scientific research, the primary incentive for participants to install the extension was receiving a personalized report on the political diversity of their Twitter friends and their curated *feed*. This report was sent to volunteers once a sufficient amount of data had been collected. Prior to the data collection process, participants were fully informed about the study’s objectives, the specific data that would be collected, and their informed consent was obtained. Following this, the extension started gathering the participants’ Twitter *feed*.

Our cohort is not representative of the Twitter audience in terms of devices usages —the data collection, performed through a desktop browser extension, is filtering out mobile users— demographics or socioeconomic status. We do not claim that our study’s findings can be extrapolated to the entire Twitter population. Nevertheless, we argue that the sanity of a platform should be maintained across devices and users’ behavior, justifying external audits —even partial ones like ours. We provide in Supplementary Information various statics regarding our cohort of participant, both demographics and on their Twitter usages. Furthermore, we present how political-group-wise, our set of participant does not significantly differ from a random sampling of Twitter French users, in terms of their friends political leaning distribution.

Taking the participants having been active on the desktop version of Twitter between March 3, 2023, and April 6, 2023 as our only objects of study, the analysis has been performed on $N=463$ participants. On average, our participants followed $682~{\scriptstyle [22,2712]}$ accounts (5-95 percentiles). In conjunction with the crowd-sourced data collection, we leveraged the Twitter API to fetch additional information, in particular the number of *tweets* published by participants’s friend within the considered timeframe. Balancing the server-side data collection burden while encompassing a large portion of the participants’ friends, we restricted our data collection to the 42k accounts followed by at least two participants, within the pool of 182k unique accounts followed by at least one participants. This subset of accounts represent on average 61.8% of the participants’ friends and cover 90.2% of *tweets* authored by friends impressed on participants’ timelines. The accounts under consideration have a median weekly *tweet* count of three, we present the cumulative distribution function of their publishing rate in Supplementary Information. Additionally, we retrieved the set of 3 million *tweets* published by the 14k accounts followed by at least three participants. We detail, in Supplementary Information, how fetched and non-fetched friends are comparable in terms of the variables of interest, such as the number of followers, activity levels, and political leanings, also to ensure the reliability of our findings, bootstrapping over participants’ fetched friends is performed for each estimate.

Finally, the partial open sourcing of the Twitter’s recommender system^9^ having revealed the importance of follow-graph-derived features^10^, we sampled the Twitter follow graph using the Twitter API and a snowball approach. Starting with our participants’ friends and a large random sample of Twitter French accounts as seeds, we fetched up to 5k followers and followees for each account. We repeated this process iteratively, including the newly fetched accounts, until we obtained more than 220k seeds. The resulting follow network consisted of 41 million nodes and 360 million edges. After pruning nodes with a degree less than 5, we were left with a follow network comprising 6 million nodes and 303 million edges. We then performed a community detection on the resulting graph, leveraging the *node2vec* algorithm^11^ for node embedding and *HDBSCAN*^12^ for the performed cluster detection. For a visual representation of the clustered network, please refer to the Supplementary Information.

### Quantification of algorithmic amplification

We defined the algorithmic amplification to measure the extent to which the *tweets* authored by a subset $G\subseteq F$ of a participant’s friends (*F*) are selected for display by Twitter’s recommender system, compared to the overall set of friends’ *tweets*. We adopted a formulation equivalent to the one of Huszár et al.^2^ but considering the point of view of the receiver, i.e. the user, instead of the authors’. The algorithmic amplification is computed as follow:

$$\begin{aligned} a(G) = \left( \frac{N^{\text {impressed}}_{G\subseteq F}}{N^{\text {published}}_{G\subseteq F} \times a_F} - 1\right) \times 100\% \end{aligned}$$

Here, $N^{\text {impressed}}_{G\subseteq F}$ represents the number of messages published by accounts in the subset $G\subseteq F$ hainvg been displayed on participant’s desktop screen. $N^{\text {published}}_{G\subseteq F}$ denotes the total number of messages published by accounts in the subset $G\subseteq F$. $a_F=N^{\text {impressed}}_{F}/N^{\text {published}}_{F}$ is a neutral baseline, it represents the fraction of messages published by the participant’s friends having been displayed on the participant’s screen, as captured by the extension. We normalize the ratio such that an amplification value of $a(G)=0\%$ indicates that the *tweets* from accounts in $G\subseteq F$ are displayed in proportion to their representation in the pool of *tweets* published by one’s friends. An amplification ratio of $a(G)=50\%$ means that the fraction of *tweets* appearing in the timelines, authored by accounts in $G\subseteq F$, is 50% higher than the fraction they represent in the pool of messages authored by one’s friends. Because *retweets* typically involve two distinct accounts, the attribution of amplification is challenging, we excluded them from the computation of the algorithmic amplification, as did Huszár et al.^2^. *Retweets* composed $11.2\%~{\scriptstyle [9.4,13.0]\%}$ of participants’ timelines.

To distinguish between the effect of the recommender system and the effect of considering only a subset $G\subseteq F$ of accounts when computing the algorithmic amplification, we compute the algorithmic amplification associated to random subsets $\tilde{G}\subseteq F$ of same cardinality ($|\tilde{G}\subseteq F|=|G\subseteq F|$); Mann-Whitney U tests between the bootstrapped amplification distributions assess the significance of the algorithmic amplification *a*(*G*). Additionally, our data collection coverage being partial, we performed bootstrapping over participants’ friends to generate robust estimates at the participant level and a bootstrapping over participants to derive robust collective measures. We provided 95% bootstrap confidence intervals for all amplification measures. Finally, we define the algorithmic amplification of tweet-related features analogously, substituting $G\subseteq F$ with the set of *tweets* published by accounts in *F* that exhibited a particular characteristic, such as a high engagement rate or being “toxic”.

Algorithmic amplification can be computed session-wise, by comparing the set of *tweets* displayed during a specific session with the recent content posted by one’s friends. However, this approach may obscure the potential consequences arising from the preference for recent content; three-fourths of the content displayed within participants’ timelines is less than 12 h old. Because, such preference for recency is an arbitrary heuristic, it is essential to consider it when evaluating how algorithmically curated timelines differ from the content produced by one’s friends. Consequently, we opted to aggregate the *tweets* displayed on participants’ screens over two weeks and compare them with their friends’ publications.

Twitter’s “For You” timelines features *tweets* send by both friends (in-network) and account one does not follow (out-of-network)^9^. The proportion of out-of-network *tweets* varies among users and has been observed to be approximately 37% for our cohort of participants. The user having actively decided to follow only their friends, we restrict the computation of the algorithmic amplification to them, where the set of *tweets* published by one’s friends serve as a natural baseline. For out-of-networks *tweets*, in the absence of a neutral baseline the algorithm could “amplify”, we will simply compare and report the distributions of the variable of interest over in-networks *tweets*, out-of-network *tweets* and over the entire set of one’s friends’ *tweets*.

#### Toxicity and sentiment analysis of *tweets*

We investigated the algorithmic amplification of *tweets* either sentimentally valenced or deemed “toxic”, such as *tweets* featuring insults, threats, and obscenities. The sentiment analysis was conducted using the XLM-T model, a multilingual language model trained on *tweets*. For the identification of toxic *tweets*, we leveraged Detoxify^13^, an open-source natural language processing model trained on Google’s Jigsaw toxic comments database. Particular emphasis was placed on the mitigation of unintentional biases in the training process, including racial biases, as discussed by Davidson et al^14^. The validity of employing this two models for this study was established by manually annotating randomly sampled *tweets* from the collected pool.

#### Account political orientation

We consider the algorithmic amplification of friends’*tweets* as a function of the political leaning of their authors. The estimation of political orientations was conducted using the Politoscope database^15,16^, which has collect all *tweets* containing French political keywords or authored by French political figures since 2016, gathering more than 700 million *tweets*.

Shortly, the French political landscape is characterized by a multipolar structure, with the current French president occupying a centrist position, as displayed in the Supplementary Information. The opposition consists of a broad left coalition on one side and a far-right group on the other. Notably, anti-system activists play a role in bridging the divide between far-left and far-right militants, resulting in a circular political landscape^17^. For instance, an account with a far-left leaning may exhibit stronger affinities with far-right content rather than with centrist content, it is then crucial to consider this circularity when analyzing potential patterns of amplification.

To determine the political leanings of Twitter accounts, we leveraged the *retweet* graph associated with political *tweets* published in 2022 (during which the French presidential and legislative elections occurred). The network of *retweet* is made of 1.2 millions nodes, 26 millions edges, where two nodes are tied if one *retweeted* the other —in a political context— at least twice. The collection procedures for this dataset are detailed in Gaumont et al.^16^, *retweets* were shown to be reliable indicators of ideological alignment. We employed the node2vec algorithm^11^ to generate embeddings of the nodes, capturing the underlying structure of the *retweet* network. Kojaku et al^18^ shown that such approach can indeed capture the community structure down to the theoretical community detectability limit. Subsequently, we calculated the angular similarity in the latent space between each account embeddings and the ones of the French political figures: Jean-Luc Melenchon [far-left], Emmanuel Macron [center] and Marine Le Pen [far-right]. Based on these similarities, we assigned a numerical political leaning to each account, ranging from -1 (for left-leaning accounts) to +1 (for right-leaning accounts); supporters of the current French President Emmanuel Macron cluster around zero. The numerical scale aligns with both the political group of members of parliament and the assessment of political experts (2019 Chapel Hill Expert Survey^19^), as well as a clustering analysis^16^. The computation details and validation tests are provided in the Supplementary Information.

## Results

### Twitter algorithmic curation increases authors’ representation inequality

As a first, high level, illustration of the shaping power of the recommender on what is seen by Twitter users, we evaluated the Gini coefficient over the number of *tweets* published and impressed by participants’ friends. We find that despite an already highly unequal situation (Gini coefficient of friends’ publications of $.72~{\scriptstyle [.56,.87]}$) in which the 10% most active participants’ friends publishes more than half of the entire set of participants’ friend *tweets*, Twitter’s recommender system increases the Gini coefficient by 14% on average (Gini coefficient of friends’ impression of $.83~{\scriptstyle [.59,.99]}$). The *tweets* from the 10% most shown friends represent more than 70% of participants’ friends impressions in their timelines. We display in the Supplementary Information the associated Lorenz curves.

**Figure 1:**
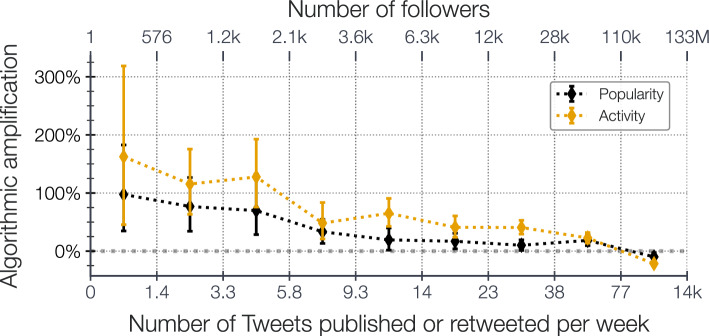
Algorithmic amplification of accounts depending of their number of followers, and of their activity in terms of number of *tweets* published or *retweeted* weekly (on average since the account creation), binned into deciles. Error-bars correspond to 95% bootstrap confidence interval of the amplification. The bold line corresponds to zero amplification.

### Small and quiet accounts benefit from higher algorithmic amplification

As displayed on Fig.  1, *tweets* of accounts having less than 576 followers (first decile) are amplified by $+97.5~{\scriptstyle [34.7.2,182.7]} ~\%$. Put differently, the proportion of *tweets* authored by small accounts is twice larger in the timelines than in the overall pool of messages authored by participants’ friends. Conversely, *tweets* of accounts with a number of followers larger than 110k are lessen by $-9.8~{\scriptstyle [-16.9,-2.9]} ~\%$. Similarly, *tweets* of accounts having published on average less than 1.4 *tweets* per week since their creation (first decile) are significantly amplified when they do publish, with an amplification of $+162.4~{\scriptstyle [45.5.2,318.7]} ~\%$. The *tweets* of highly active accounts, more than 10 *tweets* per day on average, are lessen by $-21.8~{\scriptstyle [-25.3,-17.9]}~\%$. We display in the Supplementary Information the number of followers/tweets distributions associated to out-of-networks *tweets*. For 85.5% of the participants, the number of follower distribution of out-of-networks author is stochastically smaller than for participants’ set of friends.

### Algorithmic curation affects the political landscape

**Figure 2:**
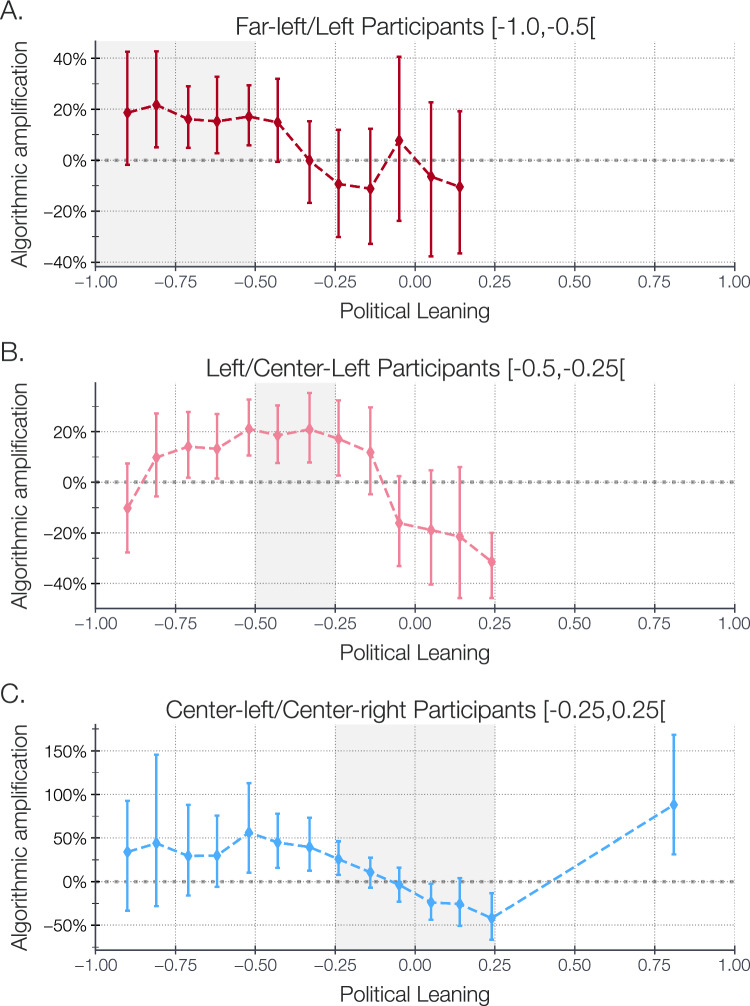
Algorithmic amplification of accounts depending of their political leaning (aggregation windows of 0.2, with successive half overlap), segmenting participants by political orientation. Error-bars correspond to 95% bootstrap confidence interval of the amplification. We shade the range of participants’ opinion for each political leaning. The bold line corresponds to zero amplification. Only statistically significant points are displayed.

After having estimated the political leaning of participants’ friends by analyzing their re*tweets* of political content, we segmented the participants based on their own political orientation; as self-declared through a form crossed with their Twitter friends orientation. Our findings reveal that for participants leaning towards the far-left (N=51) and left/center-left (N=92), Twitter’s recommender system amplifies ideologically aligned friends, see Fig. 2A,B.

As display on Fig. 2A, for far-left participants, the messages published by far-left friends are amplified by $+21.8~{\scriptstyle [5.0,42.6]} ~\%$. The amplification decreases as the opinion difference increases, until it reaches $-11.2~{\scriptstyle [-32.8,12.4]} ~\%$ for right leaning accounts’ *tweets*. Similarly, for left/center-left participants the *tweets* stemming from further-left or from right-leaning friends are algorithmically lessen, respectively by $-10.2~{\scriptstyle [-27.7,7.4]} ~\%$ and $-31.4~{\scriptstyle [-45.7,-19.9]} ~\%$, while *tweets* from ideologically aligned friends are amplified $+21.1~{\scriptstyle [10.6,32.6]} ~\%$, see Fig. 2B. Interestingly, for center-right participants (N=33), see Fig. 2C, the opposite effect is noticed, ideologically aligned accounts *tweets* are lessened by $-23.8~{\scriptstyle [-43.9,-2.6]} ~\%$, while far-left and further right *tweets* are highly amplified, respectively by $+44.1~{\scriptstyle [-28.1,145.6]} ~\%$ and $+88.0~{\scriptstyle [31.3,168.0]} ~\%$. We assessed the statistical significance of the different amplification patterns, across participants’ political leaning, through permutation tests. We have an insufficient number of far-right participants to derive meaningful statistical insights, once an adequate number of participants is obtained, we will conduct an extended analysis to explore this group further.

In Supplementary Information, we present the political leaning distribution associated with out-of-network authors’ tweets. The out-of-network political leaning distribution exhibits a greater diversity, with a variance $32.3\%{\scriptstyle [31.8,33.0]\%}$ higher than the one associated to participants’ friends. Moreover, this distribution deviates more from the political leaning distribution of participants’ friends compared to the in-network distribution, evidenced by the associated Wasserstein distance being $2.0{\scriptstyle [1.8,2.3]}$ times higher. As observed for in-network tweets, we note that out-of-network tweets tend to exhibit ideological alignment for far-left and left/center-left participants. In contrast, center-right participants tend to have a higher proportion of left-leaning and far-right tweets in their out-of-network timelines compared to their subscriptions. Additionally, a consistent trend is observed across the three examined political leanings: the prevalence of far-right tweets is notably higher in out-of-network tweets in comparison to participants’ subscriptions, respectively being $4.2~{\scriptstyle [4.0,4.5]}$ (far-left participants), $3.7~{\scriptstyle [3.1,4.3]}$ (left/center-left participants), and $1.9~{\scriptstyle [1.8,2.1]}$ (center-left/center-right participants) times higher.

### Algorithmic curation prevents diversity

The proportion of *tweets* impressed in the timelines, stemming from friends belonging to the same community (the same cluster in the network of follow) is significantly greater than in the overall pool of published messages. On the other hand, *tweets* authored by accounts from different communities are fairly displayed. This behavior holds when considering a variety of community detection resolutions, namely by changing the smallest size grouping we consider as a cluster in *HDBSCAN*^12^. When considering a minimum cluster size of 100, we detect 352 communities in our follow graph, only 4.1% of our participants’ friends belonged to the same cluster as the participant; the *tweets* stemming from this small fraction of friends are shown twice as much as, in proportion, in the timelines than what they represent in the pool of friends’ messages ($+100.2~{\scriptstyle [43.6,175.4]} \%$). The algorithmic amplification decays as the clusters are getting larger; when considering a minimum cluster size of 600, we detect 82 communities, 10.8% of our participants’ friends belonged to the same cluster as the participant and their *tweets* are amplified by $+40.8{\scriptstyle [17.9,67.6]} ~\%$. We present in the Supplementary Information the amplification for various intermediary community detection resolutions.

### Algorithmic curation amplifies toxic and sentimatally valence *tweets*

The fraction of toxic *tweets* (insults, threats, or obscenities) published by participants’ friends is approximately 2.2%. During the period 14/01/23-07/02/23, the proportion of toxic *tweets* in participants’ timelines is $+48.7~{\scriptstyle [37.6,60.8]} ~\%$ higher compared to the overall pool of messages published by participants’ friends. It is important to note that there is considerable variability in the amplification among participants, with some individuals being exposed to more than twice the proportion of toxic *tweets*. However, there is no significant correlation between the amplification at the participant level and the proportion of toxic *tweets* published by their friends. It is worth mentioning that platform-wide, toxic *tweets* receive more than twice the number of replies and likes per impression compared to non-toxic *tweets*, and experience only a 10-20% decrease in re*tweets* and quotes per impression.

Sentimentally valenced *tweets*, labeled by XLM-T^20^ as either positive or negative, experience amplification of $+2.0~{\scriptstyle [-0.3,4.4]} ~\%$ and $+5.8{\scriptstyle [3.5,8.1]} ~\%$, respectively, while neutral *tweets* are reduced by $-8.7{\scriptstyle [-11.5,-5.9]} ~\%$. During the period from December 9, 2022, to January 9, 2023 (N=101), toxic *tweets* were amplified by $+32.0~{\scriptstyle [21.7,42.7]} ~\%$ (the statistical significance of these differences was confirmed through Mann-Whitney U tests).

### Algorithmic curation alters the portrayed *tweets* popularity

**Figure 3:**
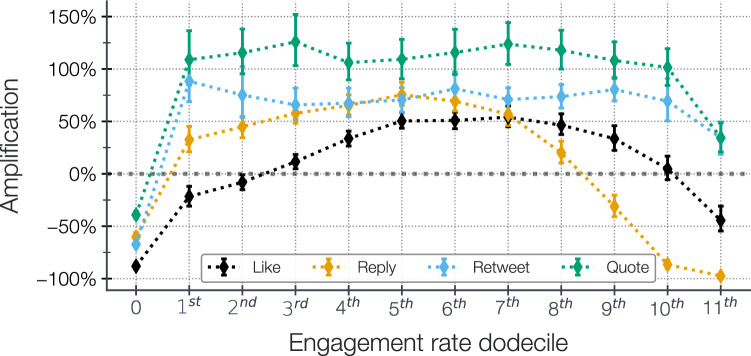
Amplification of *tweets* depending of their of engagement rate (B). We display the amplification for *tweets* having no engagement and binned in dodeciles the remainging engagement rates. Error-bars correspond to 95% bootstrap confidence interval of the amplification. The bold line corresponds to zero amplification.

Figure 3 illustrates the amplification of *tweets* published between 14/01/23 and 07/02/23, based on their platform-wide engagement rate calculated weeks after their publication to ensure metric stability. We report in the Supplementary Information*tweets* statistics and the engagement rate for each quantile. We observe distinct patterns based on different types of engagements.

First, *tweets* with no engagements are significantly quieten, with an amplification of $-88.1~{\scriptstyle [-90.5,-85.6]} \%$ for null like rate *tweets* and $-39.2{\scriptstyle [-42.2,-36.2]} ~\%$ for null quote rate ones. For *tweets* with quote and re*tweet* engagements, the amplification remains relatively constant at around $110\%$ and $75\%$, respectively, for the first ten dodeciles. However, it decreases to $34.2~{\scriptstyle [18.9,49.5]}~\%$ and $34.1{\scriptstyle [20.9,48.6]} ~\%$ at the eleventh dodecile. Notably, the algorithmic amplification is more sensitive to the like and reply rates. The amplification increases with the engagement rate, reaching a peak at the fifth dodecile for like rate and the seventh dodecile for reply rate. In the last dodecile, which corresponds to *tweets* with reply rates higher than $1.67\%$ and quote rates higher than $2.83\%$, the amplification is significantly reduced. *tweets* with high reply rates experience a decrease of $-97.4~{\scriptstyle [-98.5,-96.1]}~\%$, while *tweets* with high quote rates see a decrease of $-44.5{\scriptstyle [-54.6,-30.8]} ~\%$.

## Discussion

The partial open-sourcing of Twitter’s recommender systems revealed a convoluted blend of deep-learning models and hand-crafted heuristics^21^. For example, Twitter incorporates mechanisms such as relevance decay based on the age of tweets and the assignment of reputation scores to user based on their number of followers and a variant of the PageRank algorithm. Twitter inclination to amplify *tweets* authored by small or usually quiet accounts may then been seen as an attempt to diversify users’ *feeds*, preventing them from being dominated by spam or overly popular content. While this approach gives every user an opportunity to be heard, it also raises concerns about potential astroturfing practices, where individuals artificially boost their online presence through numerous small, fake accounts.

At the level of individual *tweets*, it’s reasonable to hypothesize that toxic *tweets* are favored by the recommender system due to their higher engagement rates in terms of replies and likes, leading to high amplification of such content. However, it’s noteworthy that, despite the recommender’s focus on engagement, popular *tweets* eventually cease to be recommended. This phenomenon may be attributed to Twitter’s design, which prioritizes the promotion of new content and sustains user engagement by favoring recent discussions. This balance between promoting popular content and encouraging a diverse range of recent content can result in situations where highly popular *tweets* are no longer recommended.

Additionally, we observe that Twitter’s recommender system tends to favor *tweets* from accounts within the same community as the user. Accounts within these communities often share common interests^9^, a characteristic leveraged by Twitter in the recommendation process^10^. It’s important to note that our intention is not to take a normative stance on the value of exposure to diverse viewpoints, as an extensive literature has comprehensively explored this topic^22–24^. Instead, we highlight that algorithmic amplification may run counter to users’ choices, if not by concealing “dissonant” content, by overwhelmingly amplifying consonant one. Similarly, our analysis reveals that Twitter’s recommender system presents a political landscape different from the one users actively subscribed to. Considering the overall objective function of Twitter’s recommender system, we hypothesize that these patterns of amplification are the one found to maximized user engagement. One could argue that the observed deviations between user subscriptions and displayed content align with user preferences. However, Milli et al’s recent study revealed that users were less likely to prefer the political tweets selected by engagement-based algorithms compared to reverse chronological timelines^7^, highlighting the disparity between stated and revealed preferences.

Our audit underscores the systemic effects of Twitter’s recommender system on the information landscape portrayed to users, resulting in more toxic timelines and affecting the mutual representation of political groups. Additionally, the amplification of small accounts can render the digital space more susceptible to manipulative practices like astroturfing. Nonetheless, recommender systems remain intricate entities with numerous features and data points, warranting further investigations to unravel their complexities. Various confounding factors are at play, and our audit captures only a fraction of the resulting characteristic in the final end product. While enhancing the transparency of recommender system designs, such as through open-sourcing algorithms, may offer insights into their internal mechanisms and skewed suggestions, independent audits equipped with access to large-scale data will remain indispensable for the regulation of digital services, as specified by the 40th article of the European law on digital services^25^.

### Supplementary Information

Supplementary Information.

## Data Availability

We adhere strictly to both Twitter’s developer policy and Horus’s privacy policy. As per these policies, we offer aggregated data upon request from the corresponding author, to enable the reproduction of our Figures.
