# Supplementary material for: Crowdsourced audit of Twitter’s recommender systems
Source: Sci Rep. 2023 Oct 5;13:16815. doi: 10.1038/s41598-023-43980-4 (PMC10556069; doi:10.1038/s41598-023-43980-4)
Supplement: Supplementary file 1 — Supplementary Information. [file 41598_2023_43980_MOESM1_ESM.pdf]

Supporting Information for

Crowdsourced Audit of Twitter's Recommender Systems

Paul Bouchaud, David Chavalarias, Maziyar Panahi

Corresponding Author Paul Bouchaud.
E-mail: paul.bouchaud@iscpif.fr

This PDF file includes:

Figs. S1 to S10
Tables S1 to S2
SI References

1. Participants Statistics

A. Demographic. The proportions of participants in different age groups are as follows: 16.5% of the population is aged ≤ 25, 27.0% falls within the 25-34 age range, 37.8% are aged 35-49, and the remaining 18.8% are ≥ 50 years old. 86.7% of the participants declared themselves as "man", 13.3% as "woman" and less than 0.1% as other.

B. Twitter Usage. During the data collection timeframe, the participants connected to Twitter on their desktop on average 5 times a day ($[1, 15]$ 5-95 percentiles), for a median session length of 3 minutes ($[0.8, 35]$ 5-95 percentiles). During each session, the participants read on average 30 tweets, see the distribution on Figure S1.

C. Political Representativeness. To assess the representativeness of our participant cohort with respect to the political leanings of the accounts they follow, we conducted the following methodology: We randomly selected French Twitter accounts from the follower network and determined their political leaning (far-left, left, or center) based on the political leanings of their friends, as for our participants. Subsequently, we computed the political leaning distribution of the friends of these accounts. Following this, we calculated the Wasserstein distance between the overall political leaning distribution and the distribution generated by the political leanings of friends of a random subset of users (matching the cardinality of participants) of a given label, either far-left, left, or center. The Wasserstein distance has been computed taking into account the periodicity in the opinion space at ± 1. Finally, we determined the Wasserstein distance between the overall political opinion distribution and the distribution of political leaning of participants' friends, restricted to participants of a specific leaning. Overall, we fail to reject the hypothesis according to which the distribution of political leaning among participants' friends significantly differs from the one derived from a random sampling of French Twitter users with corresponding political leanings.

2. Effect of incomplete data collection

To rule out the potential influence of incomplete data collection on the observed disparity between content displayed in participants' timelines and their friends' posts, we conducted tests on the distribution of key variables: followers count, *tweet* count, and political leanings. We compared these distributions between friends whose data was and wasn't collected.

Regarding followers count and *tweet* count, we employed chi-square tests to assess frequency differences in respective bins between the two groups. Our findings indicate that, at a significance level of 0.05, 94.5% (95.5%) of participants exhibited no statistically significant disparities in their followers count (tweet count) between fetched and non-fetched friends.

For the continuous variable of political leaning, we performed a two-sample Kolmogorov-Smirnov test to compare the political opinion distributions of fetched and non-fetched friends. In 79.4% of cases, at a significance level of 0.05, no statistically significant differences were observed between the political leanings of fetched and non-fetched friends.

In each case, we recalculated the algorithmic amplification specifically for participants where no statistical differences were found between fetched and non-fetched friends. The results presented in the main text remain consistent and unaffected by this analysis.

3. Political Determination

The estimation of political orientations was conducted using the Politoscope database as follow:
Firstly, we initialized the opinion values of the far-left (Jean-Luc Mélenchon) and far-right (Marine Le Pen) leaders as ± 0.75, (arbitrarily chosen value). Subsequently, the opinion of the centrist leader (Emmanuel Macron) was determined by taking the average of the opinions of the two anchored leaders, weighted by the angular similarities between the nodes' embeddings, obtained using node2vec(1). Interestingly, this calculation resulted in an opinion value close to zero (-0.02). The angular similarity is the complement of the angular distance which contrary to the cosine similarity is a formal distance metric: angular similarity $= 1 - \frac{\arccos(\text{cosine similarity})}{\pi}$. Then, for each Twitter account, we computed the angular similarity between the account's embedding and the embeddings of the three leaders. The political leaning of the account was then determined by averaging the opinions of the two closest leaders, considering their angular similarities as weights. In cases where the two closest leaders were the extreme ones, we accounted for the periodicity of the opinion space, ensuring that the assigned opinion spanned the entire range from -1 to +1. Furthermore, we only assigned a political leaning if the angular similarity with the closest leader was at least 10% higher than the similarity with the farther leader. Accounts without a clearly defined political leaning constituted less than 10% of the accounts in our database.

To validate the stability of the resulting opinion scale, we experimented different anchors, i.e. different leaders and different anchors values, leading to the similar assignments. Additionally, we observed a correspondence between the opinion scale and a cluster analysis of the *retweet* graph (2). For a visual representation of the political landscape, Figure S2 presents the spatialized *retweet* graph, where nodes are color-coded based on their assigned numerical opinion, providing a clear interpretation of the political landscape. Moreover, the political groups declared by French members of Parliament aligned with the opinion scale, as illustrated in Figure S3. The Pearson correlation coefficient between the political leaning assigned to MPs (averaged by political group) and the left-right overall ideological stance of the parties, assessed by political experts within the 2019 Chapel Hill Expert Survey(3), equals $r = 0.986, p < 10^{-5}$, as displayed on figure S4.

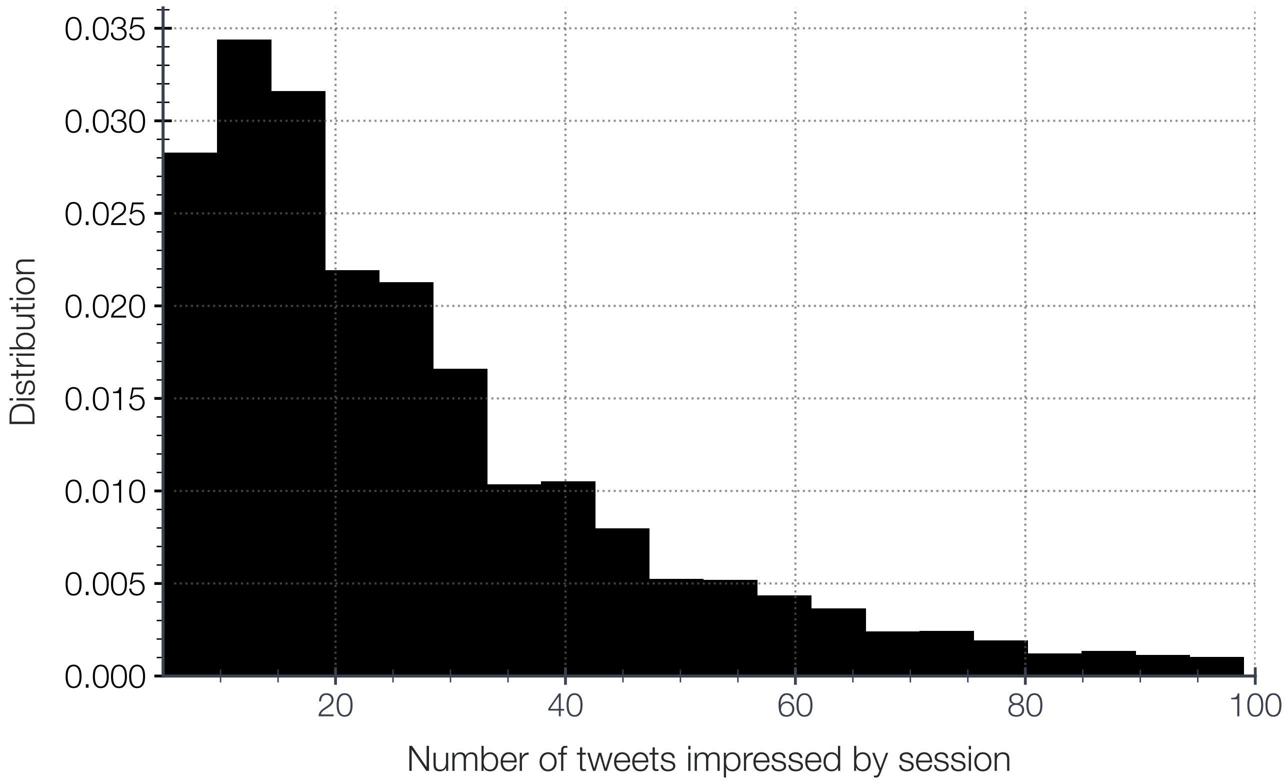

Fig. S1. Distribution of the number of *tweets* impressed by session, truncated at 100 *tweets* (2.6% of the session are in the truncated tails)

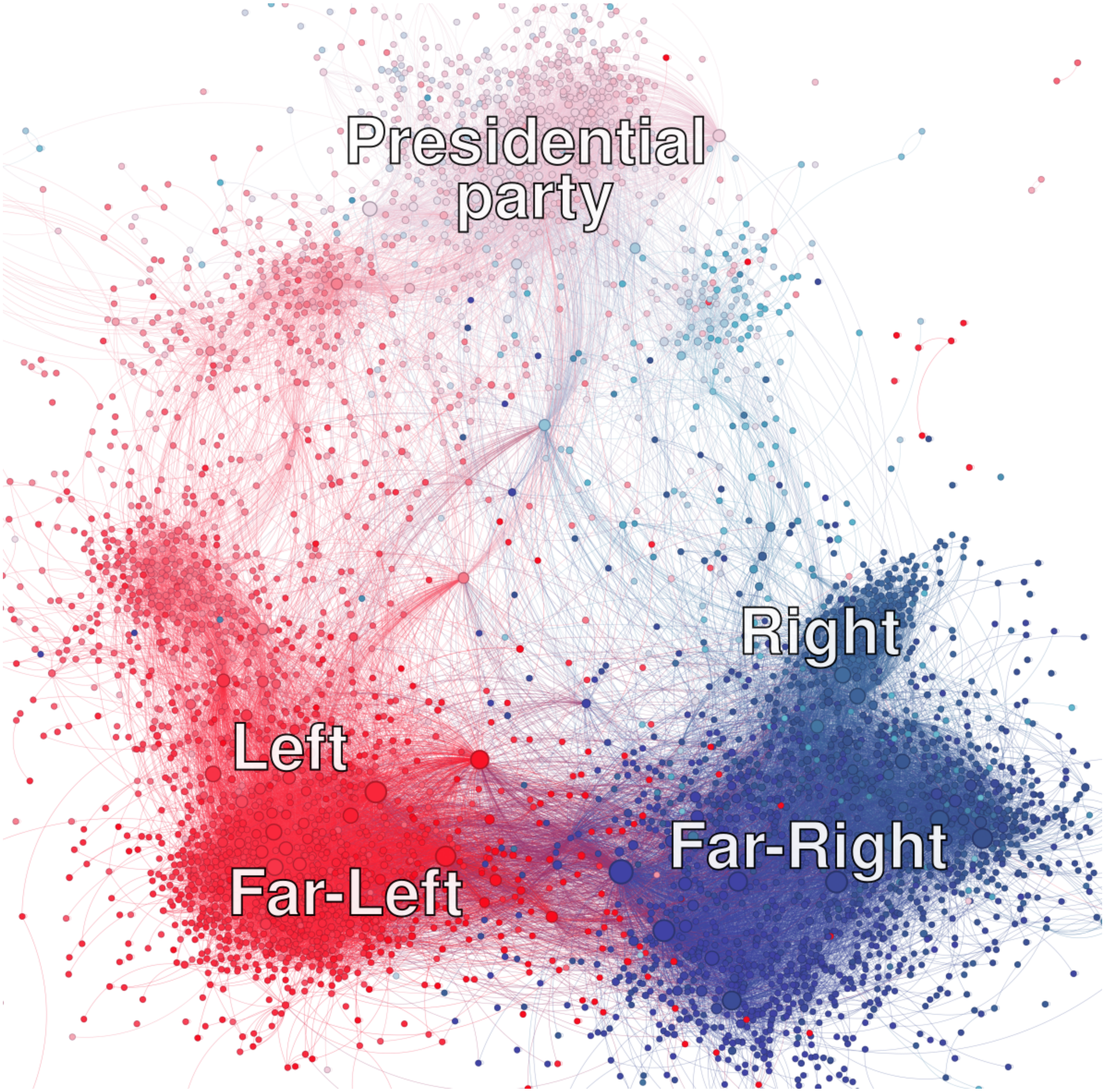

Fig. S2. Graph of *retweets* associated to political messages published during the first semester of 2023, filtering edge with a weight less than 5. Spatialized via ForceAtlas2 (4). Nodes are colored by the assigned numerical opinion.

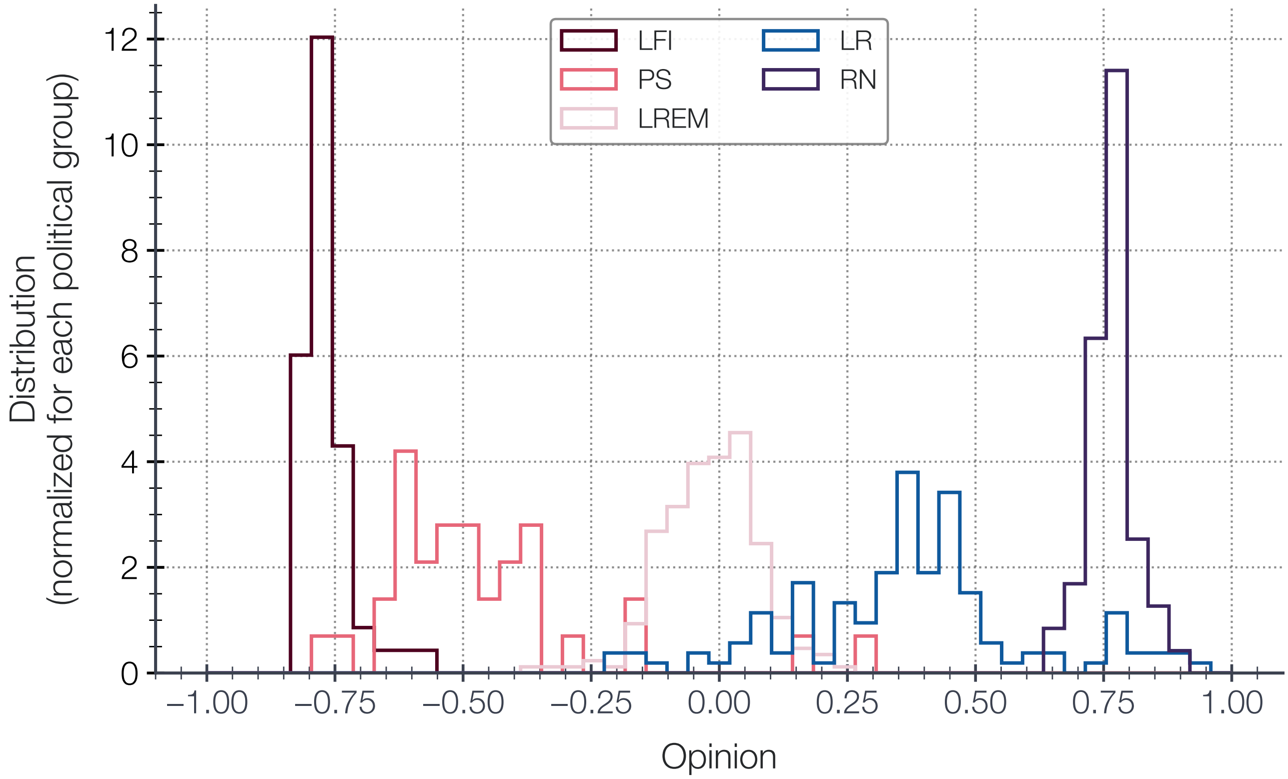

Fig. S3. Distribution of the political leaning assigned to members of French Parliament belonging to the main declared political groups, from left to right: "LFI", "PS","LREM", "LR", "RN"

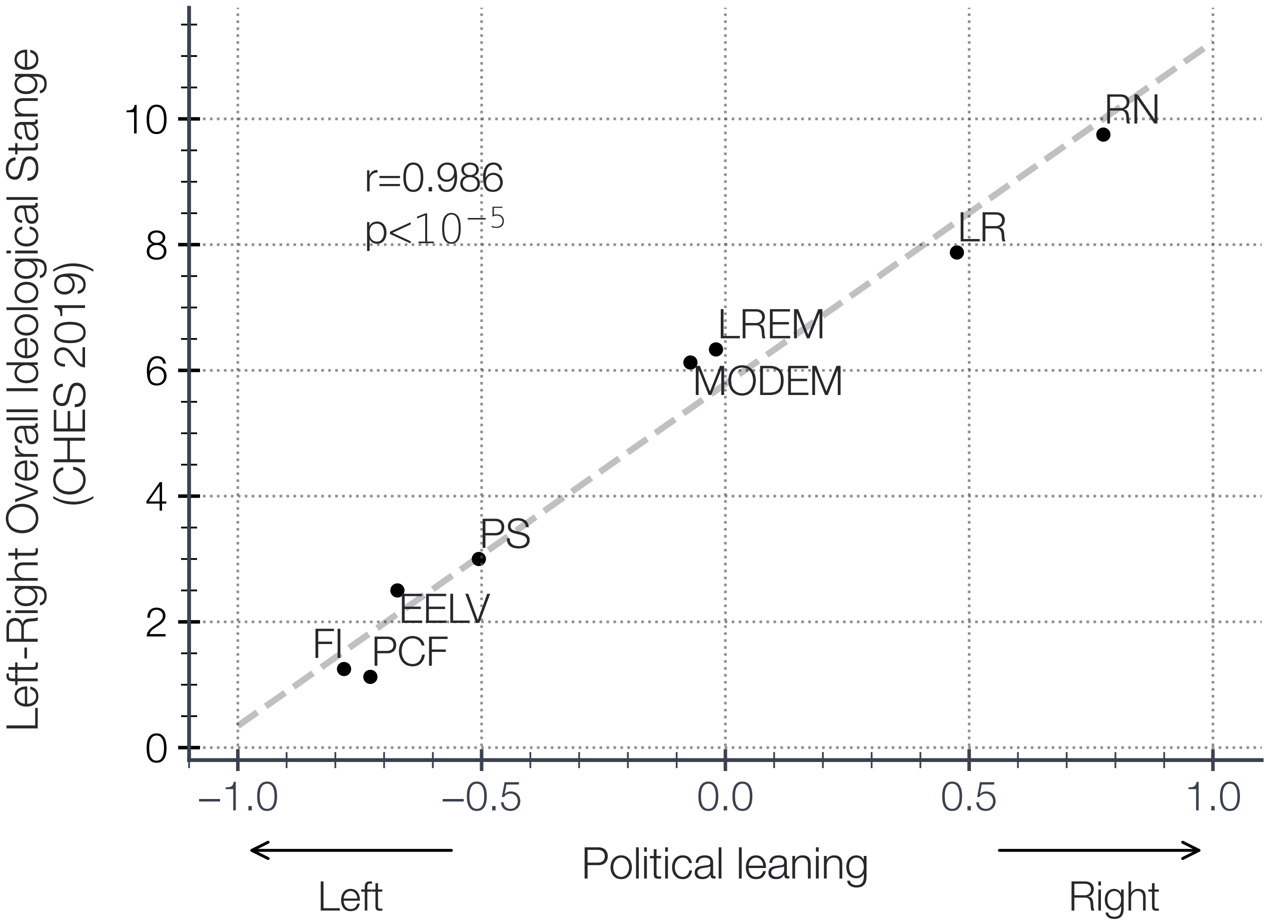

Fig. S4. Comparison between the political leaning assigned to the MPs with the described methodology and the assessment made by political experts within the 2019 Chapel Hill Expert Survey(3)

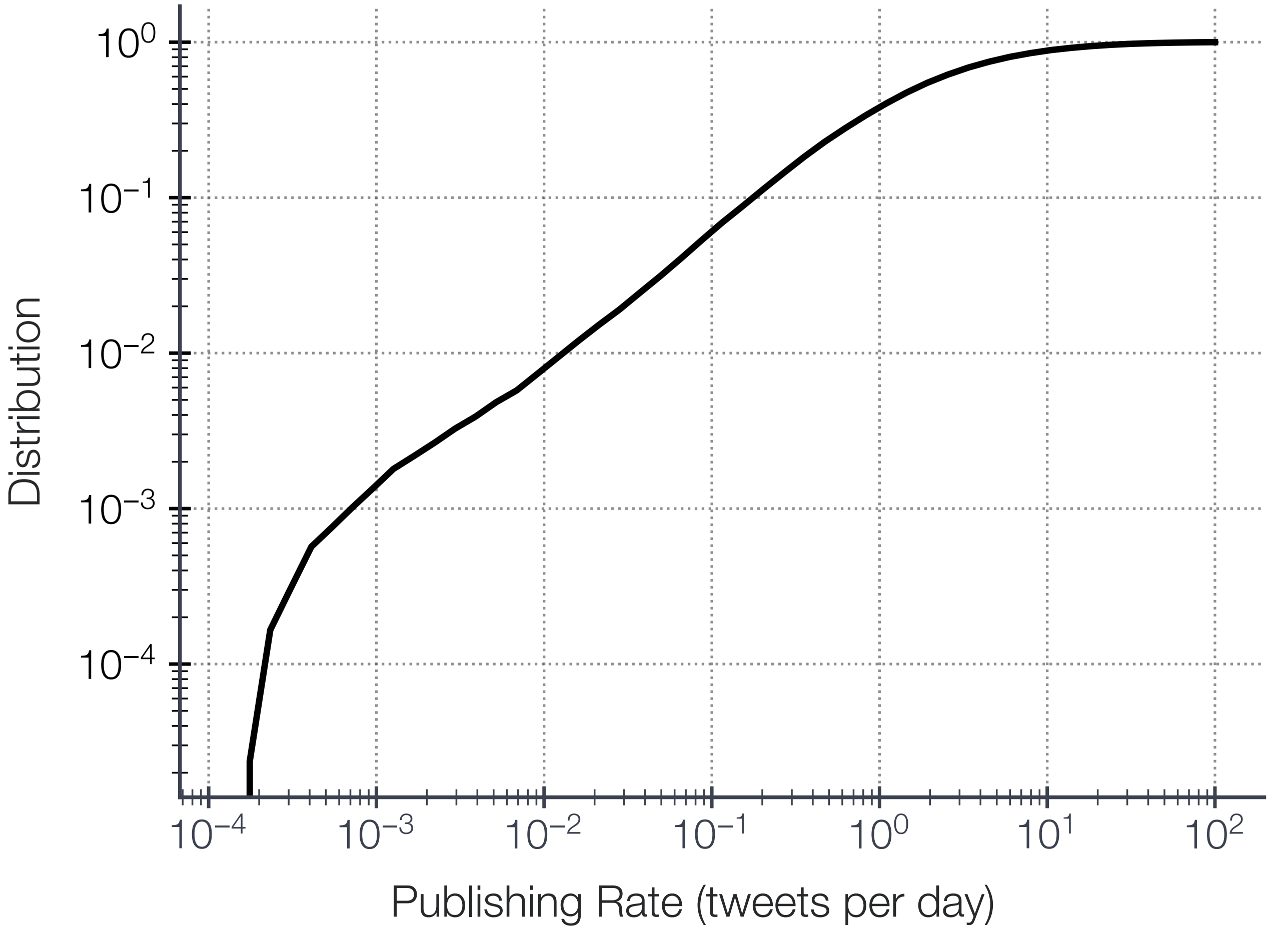

Fig. S5. Cumulative distribution function of the publishing rate of the 42k accounts considered in the analysis, in log-log scale

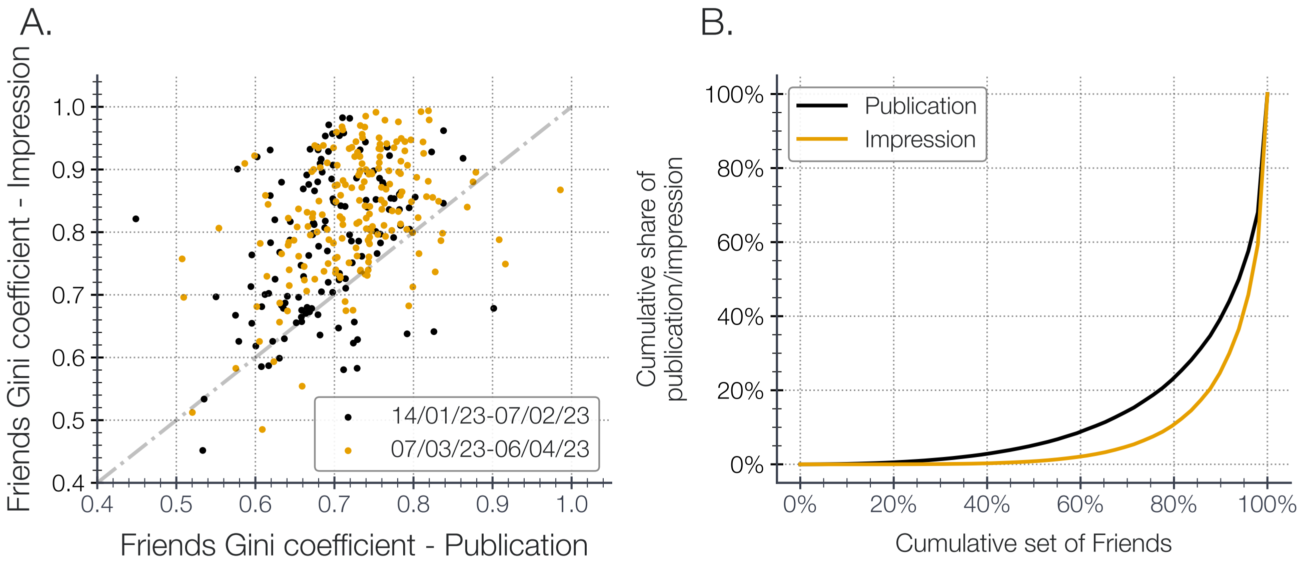

Fig. S6. (A) Gini coefficient of participants' friend published messages vs. Gini coefficient of participants' friend impressed messages. Only participants having seen more than 500 *tweets* (captured by the browser extension) during the consider period are displayed (B) Lorenz Curves associated to the publication and impression Gini coefficients, averaged over participants.

 Paul Bouchaud, David Chavalarias, Maziyar Panahi

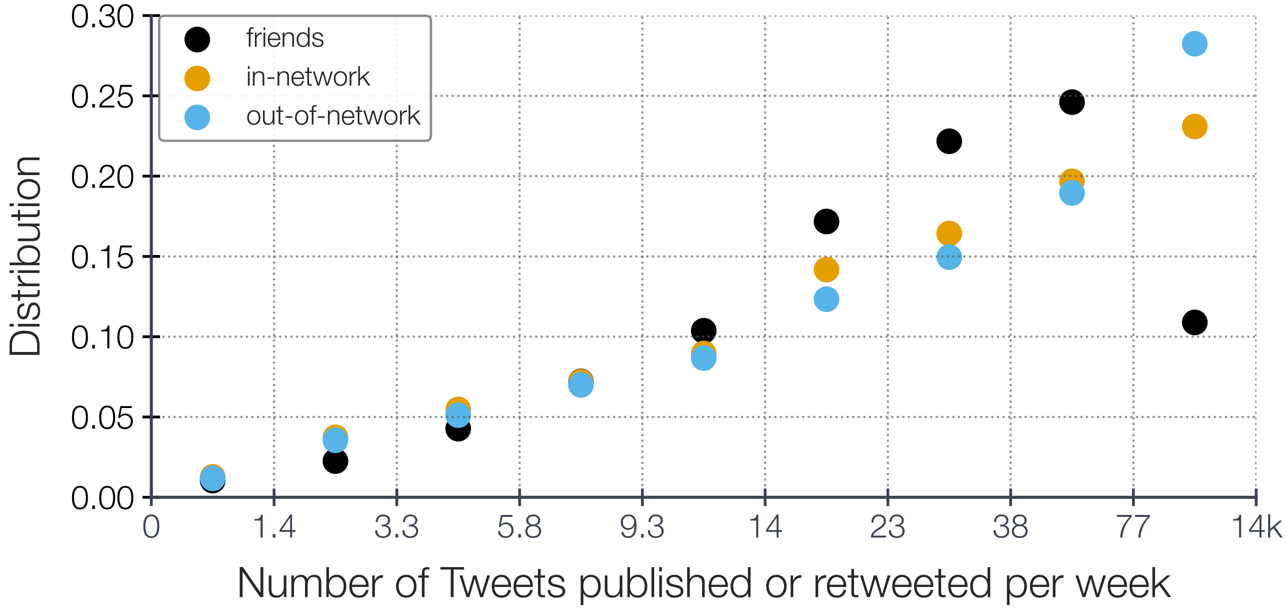

Fig. S7. Distribution of the number of *tweets* published or *retweeted* weekly (averaged since the account creation) by accounts: Followed by Participants (Black), Appearing in Participant Timelines as In-Network (Orange), Appearing in Participant Timelines as Out-of-Network (Blue).

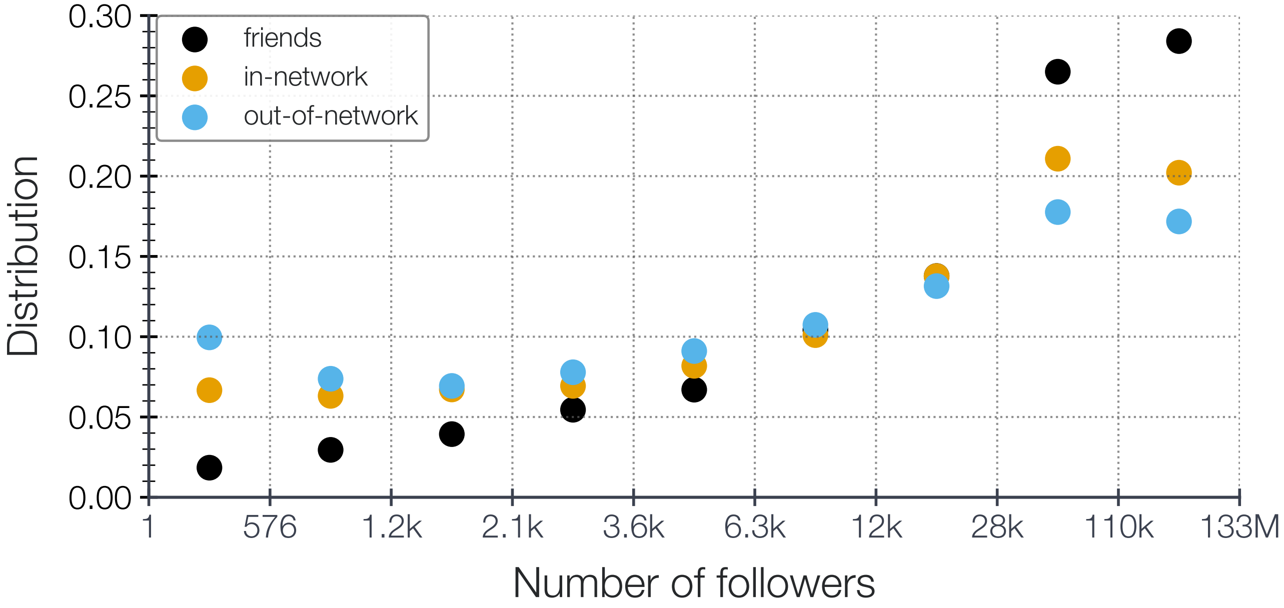

Fig. S8. Distribution of the number of followers of accounts: Followed by Participants (Black), Appearing in Participant Timelines as In-Network (Orange), Appearing in Participant Timelines as Out-of-Network (Blue).

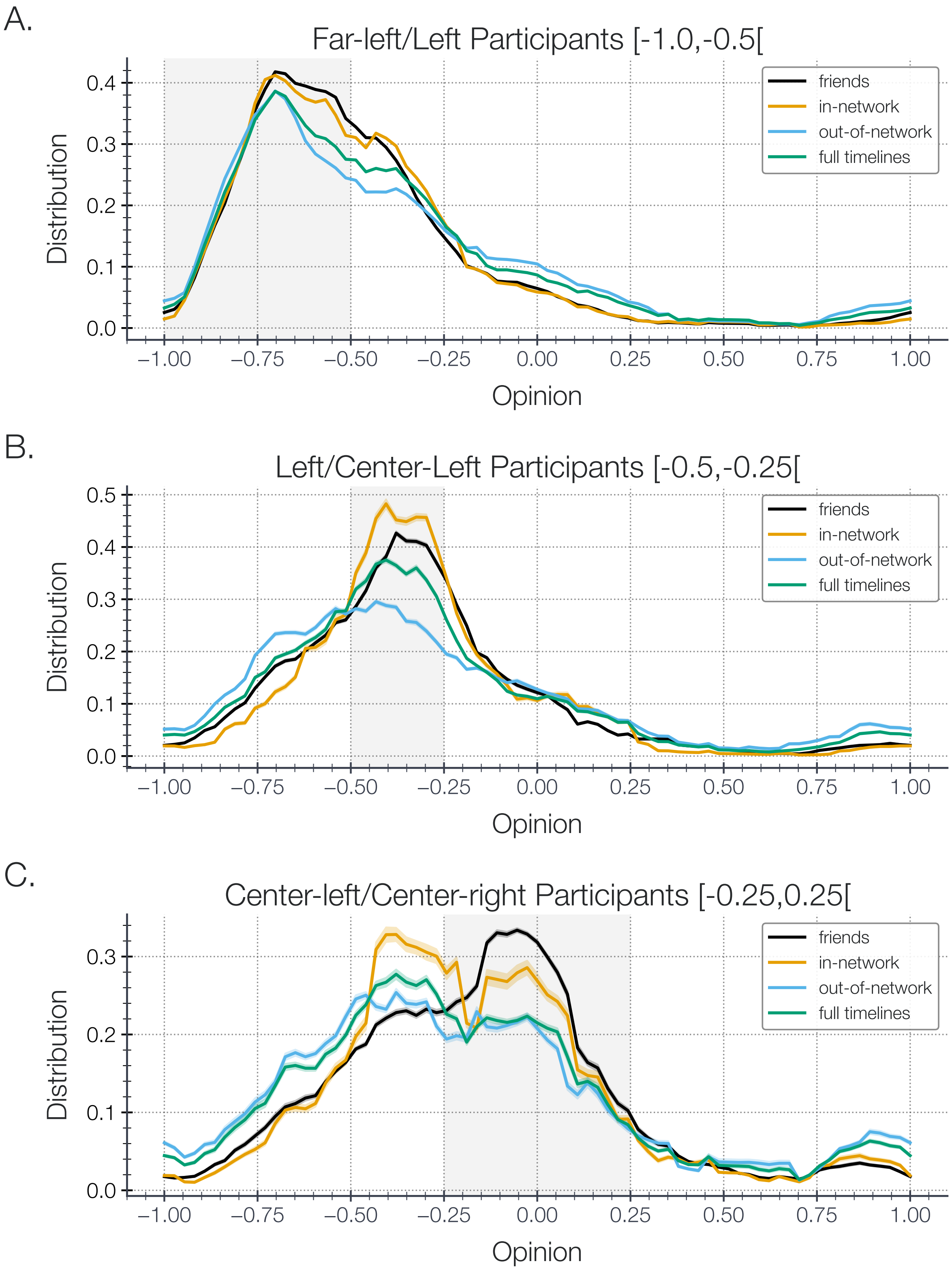

Fig. S9. Distribution of the political leaning of accounts: Followed by Participants (Black), Appearing in Participant Timelines as In-Network (Orange), Appearing in Participant Timelines as Out-of-Network (Blue), Appearing in Participant Timelines In and Out network combined (Green). We segment participant based on their political leaning. Filled area corresponds to standard error determined via bootstrap over participants.

References

1. A Grover, J Leskovec, node2vec: Scalable feature learning for networks in *Proceedings of the 22nd ACM SIGKDD International Conference on Knowledge Discovery and Data Mining.* (ACM), (2016).
2. N Gaumont, M Panahi, D Chavalarias, Reconstruction of the socio-semantic dynamics of political activist twitter networks—method and application to the 2017 french presidential election. *PLoS ONE ONE* **13**, e0201879 (2018).
3. S Jolly, et al., Chapel hill expert survey trend file, 1999–2019. *Elect. Stud.* **75**, 102420 (2022).
4. M Jacomy, T Venturini, S Heymann, M Bastian, ForceAtlas2, a continuous graph layout algorithm for handy network visualization designed for the gephi software. *PLoS ONE* **9**, e98679 (2014).

Table S1. Algorithmic Amplification for various cluster resolutions, using HDBSCAN over node2vec graph embeddings, reduced with UMAP

min_cluster_size	Number of detected clusters	Proportion of friends in the same cluster than the participant	In-Cluster friends' *tweets* amplification	Out-Cluster friends' *tweets* amplification
100	352	4.1 (2.7,5.5) %	100.2 (43.6, 175.3) %	6.5 (-0.4,14.4) %
200	173	8.3 (6.3,10.4) %	80.1 (23.1, 154.9) %	1.5 (-6.3, 9.6) %
300	129	9.1 (6.8,11.2) %	58.8 (16.8, 127.3) %	4.4 (-2.9, 13.1) %
400	110	9.4 (7.5,11.6) %	56.5 (7.3,127.6) %	3.5 (-4.6, 11.8) %
500	92	9.0 (7.0,11.2) %	50.5 (24.1, 76.7) %	3.0 (-4.0, 11.1) %
600	82	10.8 (8.8,12.9) %	40.8 (17.9, 67.6) %	2.7 (-4.6, 9.6) %

Table S2. Engagement rates by quantile, computed over our set of 3 millions *tweets* published by our participants' friends, after filtering out *tweets* without engagement (29.0% *tweets* had no reply, 48.9% no *retweet*, 71.7% no *quote* and 14.7% no *like*). Please be cautious when interpreting the *retweet* rate, a *tweet* can continue to accumulate *retweets* even without being impressed, if the *retweets* of this original *tweet* are impressed instead. As a result, the *retweet* rate in the last quantile may be misleading, nevertheless, we compare in the main text the algorithmic amplification for a given engagement type, across quantiles.

Quantile	Reply (%)	Retweet (%)	Quote (%)	Like (%)
1	0.000118	0.000122	0.000037	0.00222
2	0.00803	0.0142	0.00221	0.0961
3	0.0153	0.0248	0.00376	0.180
4	0.0253	0.0371	0.00551	0.283
5	0.0392	0.0523	0.00754	0.401
6	0.0596	0.0716	0.0100	0.543
7	0.0908	0.0970	0.0132	0.720
8	0.144	0.132	0.0174	0.952
9	0.256	0.187	0.0235	1.28
10	0.581	0.281	0.0329	1.80
11	1.67	0.492	0.0528	2.83
12	100	750	8.33	100

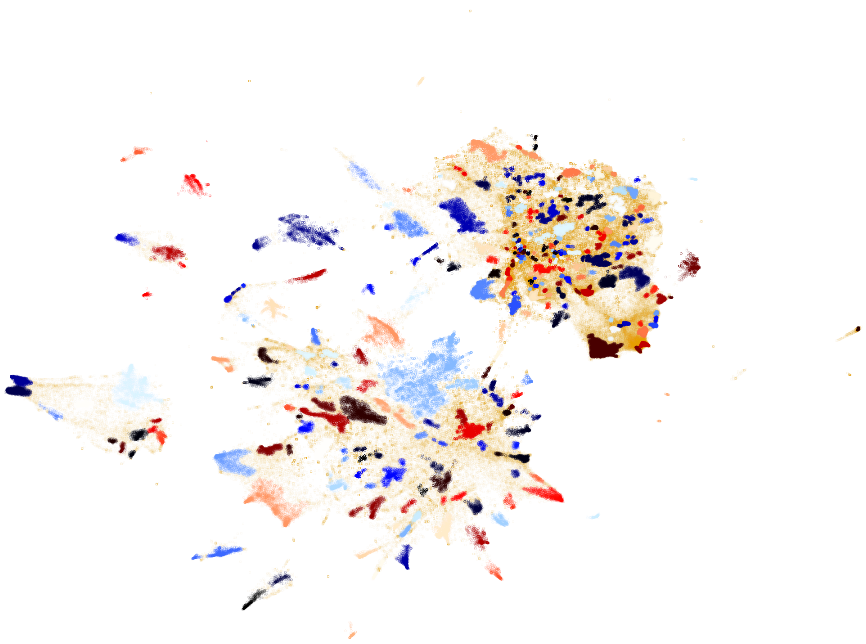

(a) Minimum cluster size 100

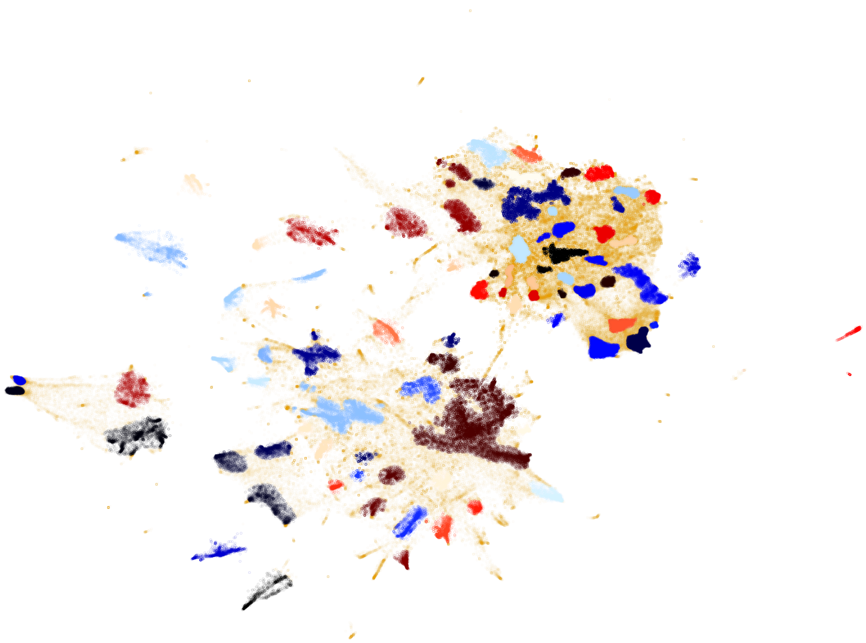

(b) Minimum cluster size 600

Fig. S10. Network of Follow, visualized after a dimensional reduction through UMAP over node2vec embeddings. For clarity sake, only participants' friends and the 220k snowballs seeds are displayed. Clusted idenfied by HDSCBAN are colored, nodes in orange corresponds to outliers, belonging to no identified clusters.
